# Benzimidazole resistance of sheep nematodes in Norway confirmed through controlled efficacy test

**DOI:** 10.1186/1751-0147-54-48

**Published:** 2012-08-29

**Authors:** Atle V Meling Domke, Christophe Chartier, Bjørn Gjerde, Snorre Stuen

**Affiliations:** 1 Norwegian School of Veterinary Science, Sandnes, Norway; 2 LUNAM Université, Oniris, Nantes-Atlantic College of veterinary medicine and food sciences and engineering, UMR BioEpAR, Nantes, F-44307, France; 3 Norwegian School of Veterinary Science, Oslo, Norway

**Keywords:** Benzimidazole, Anthelmintic resistance, FECRT, Controlled efficacy test, Sheep

## Abstract

**Background:**

Resistance against benzimidazoles (BZ) has recently been detected in Norwegian sheep flocks through a large scale prevalence survey based on the faecal egg count reduction test (FECRT). The use of this test in combination with bulk larval culture only gives an indication of which gastrointestinal nematodes genera that are involved and these results have to be confirmed by a controlled efficacy test (CET) to get accurate information about resistant nematodes populations at species level. A CET was therefore performed with larvae from two flocks where BZ resistance was previously detected through FECRT.

**Results:**

The latter test confirmed the previous results in both flocks. In flock A, the BZ resistant nematode population consisted solely of *Haemonchus contortus,* whereas *H. contortus* and *Teladorsagia circumcincta* comprised the resistant worm population in flock B.

**Conclusions:**

Some discrepancies that have been recorded between FECRT and CET results regarding time for post-treatment coproscopical examination and a temporary suppression of faecal egg excretion are discussed.

## Background

Anthelmintic resistance (AR) is currently threatening the small ruminant industry [1]. Multi-resistant nematodes occur worldwide, causing production losses and animal welfare concerns in the sheep industry [2,3]. In Norway, the prevalence of AR has recently been investigated by a large-scale survey in sheep using the faecal egg count reduction test (FECRT) [4]. Post-treatment positive coproscopical results have been recorded for benzimidazoles (BZ), mainly in the coastal area of Norway. However, as FECRTs may give false-positive results in relation with drug administration issues and does not identify the resistant species properly [5], a controlled efficacy test (CET) was performed in two selected flocks with previously reported BZ resistance both to confirm the presence of resistance and to further characterize the nematode populations involved.

## Methods

### Selection and characteristics of the tested flocks

Based on the results from the FECRT performed by Domke et al. [4], gastrointestinal nematode (GIN) populations from two of the eight sheep flocks with BZ resistance were sampled for a controlled efficacy test. Both flocks were located in Rogaland County in south-western Norway. Norwegian white sheep (NKS, Norsk kvit sau) was the dominant breed in both flocks. Both farms used mountain pastures for half of the flock, while the rest of the animals were kept at cultivated pastures close to the farms. BZ’s has been used for several years in both flocks, however AR was not suspected before the FECRT survey.

Flock A contained approximately 180 winter-feed ewes, which are housed during the winter (December – April). Flock B had a herd size of 100 winter-feed ewes and houses the animals only during the lambing period in March - April.

In flock A, the faecal egg count reductions (FECRs) in the BZ treated group were 81% and 89%, in 2009 and 2010, respectively. In flock B, the corresponding figures were 83% and 75% [4]. Flock A used four annual anthelmintic treatments of the lambs and practiced until recently “dose-and-move” (drenching and changing of pasture strategy). Flock B had six annual deworming’s of their lambs and drenched their lambs every 4^th^ week from May till August. This flock was still practicing “dose-and-move”.

The two flocks were selected on the basis of the results of the FECRTs in 2009 and 2010 and because of their proximity to the lab. However, these flocks had a similar use of pastures to the six remaining sheep flocks in Rogaland where BZ resistance had been previously detected [4].

### Controlled efficacy test (CET)

A controlled efficacy test was performed with larvae from both sheep flocks in the autumn 2011. The test was performed according to the WAAVP guidelines [5]. In each flock faeces from at least 20 ewes and 20 first year grazing lambs were sampled rectally and used for bulk coproculture. Twenty-four lambs at an age of 5–6 months that had been raised in a zero-grazing system at Høyland Field Station were experimentally infected orally with infective L3 larvae from the bulk coproculture. Twelve lambs were used for each of flock A and B, and were designated as Group A and B, respectively. The numbers of infective larvae for inoculation were 5000 and 4000 per lamb in Group A and B, respectively. The inoculum contained infective third stage larvae (L3) of both *Haemonchus* and *Teladorsagia/Trichostrongylus* types: 42/58% and 20/80% for Group A and B respectively. The lambs were inoculated by a gastric tube. The CET protocol was ethically approved by the Norwegian Animal Research Authority (by Dr. Martha J. Ulvund, FDU ref. 2011/1237).

A FEC was performed seven days before inoculation (day −7), at day of inoculation (day 0) and at day 7, 18, 28 and 35 (only Group B) after inoculation to control the establishment of the nematode infection. When the infections had been established with a faecal egg excretion of at least 150 eggs per gram faeces (EPG) in each lamb, the 12 lambs in each group were randomly assigned to two subgroups of six animals. One subgroup (A1, B1) received 3.8 mg/kg albendazole (Valbazen vet.®Pfizer) orally, whereas the other subgroup (A2, B2) served as untreated control group. The anthelmintic treatment was performed at day 28 and day 35 post inoculation for subgroup A1 and B1, respectively.

All the lambs were euthanized eight or nine days after treatment by intravenous injection of 10% barbiturate (Pentobarbital 10%, NAF) and examined post-mortem.

The abomasum and small intestine were processed according to MAFF (1986). A worm count was conducted on 1/20 aliquots of the content of the abomasum and the four first meters of the small intestine. The mucosa of the abomasum and the small intestine were carefully washed to recover all worms. The large intestine was opened and the contents carefully examined and all living worms were collected. The recovered worms were transferred to labelled bottles with 70% ethanol for preservation. All male worms from the abomasum and the small and large intestine were examined under microscope and identified on morphological traits [6,7].

### Statistical analysis

The Mann–Whitney test with significance by p < 0.005 was used when comparing the worm burden and EPG between the control and treated group.

## Results

In group A there was a significant reduction in the mean EPG of 72.6% following treatment (p < 0.005) EPG in the treated subgroup A1 (700 EPG vs 192 EPG), whereas no significant change was observed in the control group A2 (708 EPG vs 733 EPG) (Table 1). In the post-treatment coproculture of A1, *Haemonchus* was the only larval type recovered. Similarly, in the necroscopic examination, *Haemonchus contortus* was the only species identified in control and treated animals and worms of this species were detected from the abomasum from all animals. The reduction in the *H. contortus* adult population in subgroup A1 compared to the untreated subgroup A2 was 79%.

**Table 1:** Mean number (sd) of adult nematode species recovered from the abomasum from the control group and the BZ-treated group, and the reduction (in %) in number of adults and excreted eggs pre- and post-treatment

		**EPG(sd)**			**Abomasum (arithmetic mean (sd))**				
**Group**	**Treatment**	**Pre-treatment**	**Necr. Ex**	**Reduction (%)**	**Total (arithmetic mean (sd))**	***T. circumcincta***	**Reduction (%)**	***H. contortus***	**Reduction (%)**
A	Control	708 (±120)	733(±258)	0	143(±1000)			143(±100)	
	BZ	700(±247)	192(±146)	72.6	30(±20)			30(±20)	79
B	Control	425(±281)	508(±656)	0	126(±82)	33(±19)		97(±74)	
	BZ	533(±205)	0(±0)	100	80(±33)	45(±17)	0	63(±31)	35

In group B, there was a significant reduction in the mean EPG of 100% following treatment (p < 0.005) in the treated subgroup B1 (533 EPG vs 0 EPG), whereas a slight increase in the mean EPG was noticed in subgroup B2 (425 EPG vs 508 EPG). Post-treatment, both *Teladorsagia circumcincta* and *H. contortus* worms were collected from the subgroups. Three of the lambs in subgroup B2 and four of the six lambs in subgroup B1 had *T. circumcincta* present in the abomasum. The *T. circumcincta* worm burdens were similar between B1 and B2 (45 vs 33 worms). For the *H. contortus* populations, there was a reduction of 35% in the mean worm number in subgroup B1 compared to B2 (63 vs 97 worms). *H. contortus* was found in all abomasums of all the lambs in group B. No other nematodes in the abomasum or small intestine were found in the necropsied animals.

## Discussion

This is the first report of BZ resistance in *T. circumcincta* and *H. contortus* in Norway confirmed through CET. A large scale prevalence survey on anthelmintic resistance in small ruminants in Norway in 2008–2009 using the FECRT indicated a 100% efficacy of macrocyclic lactones (ML) and a BZ resistance in 10.5% of the sheep flocks [4]. When restricting the area to Rogaland County and to at-risk sheep farms, the AR-prevalence reached 80%. The nematode genera involved in BZ resistance were identified as *Teladorsagia/Trichostrongylus* and *Haemonchus* type*,* based on L3 identification from the post-treatment coproculture [4]. The present study, using the controlled efficacy test, confirmed that the actual species involved were *T. circumcincta* and *H. contortus*, including a mixed infection in flock B. *T. circumcincta* is the most prevalent GIN in sheep in Norway (75% of farms) and is found in all parts of Norway (Domke et al. submitted). On the other hand, the blood sucking nematode *H. contortus* is also present in all regions of Norway (34% of farms)as north as Lofoten in Northern Norway, but is mainly located at the south western coastline (Domke et al. submitted). As far as the worm burdens are concerned, *T. circumcincta* and *H. contortus* are also the major parasites for sheep in Norway (mean number of 233 and 724 worms per animal, respectively) (Domke et al. submitted).

In group A, despite the occurrence of *Teladorsagia/Trichostrongylus* larval type in the inoculums, the nematode population established in the controls was only composed of *H. contortus*. Moreover the overall establishment rate of worms in experimentally infected lambs in both CET’s seemed rather low (around 3%). Gaba et al. [8] have shown high variability of establishment rates of *T. circumcincta* in sheep using a meta-analysis of 87 experiments. Amongst the main factors involved, the authors quoted the breed and age of lambs, the infection mode (single, repeated, experimental or natural), and the infective dose and suggested two hypotheses, at least for experimental conditions, related to host immune response and parasite virulence. Regarding the first hypothesis, lambs were naïve and aged 5–6 months old, which is in the range of published experiments [8]. However, it has been shown that greater FEC and worm burdens could be obtained in experimental *T. circumcincta* infections in quasi-naïve lambs concurrently immune-suppressed with methylprednisolone acetate [9]. Regarding the second hypothesis focusing on parasite virulence and infectivity, it cannot be further documented in our conditions a possible interaction between *Teladorsagia*/*Trichostrongylus* and *Haemonchus* during the host establishment as described by Hoste and Cabaret [10]. Otherwise, inoculums sizes around 7,500 to 14,000 L3 in mixed infections could have probably increased the final adult population [5].

In the treated subgroup B1, the faecal egg excretion was nil nine days after treatment, whereas adult *T. circumcincta* and *H. contortus* worms were recovered in the necropsied lambs. A temporary suppression of egg excretion by worms surviving the treatment may be expected. This phenomenon has been mentioned in particular after ML or BZ treatment and a time lag longer than eight days is recommended for BZ evaluation in FECRT [11]. A longer interval of ten days post treatment has even been suggested [12].

## Conclusions

The CET’s confirmed in two flocks the results from the earlier conducted FECR tests. *H. contortus* and *T. circumcincta* are the two species involved, alone or in combination, as BZ resistant worms in these sheep flocks located in Rogaland County.

## Competing interests

The authors declare that they have no competing interests.

## Authors’ contributions

AVMD, CC, BG, and SS initiated and designed the study. AVMD performed the CET and the statistical analysis. AVMD and SS drafted the manuscript. All authors read and approved the manuscript.
